# Translating insights from neuropsychiatric genetics and genomics for precision psychiatry

**DOI:** 10.1186/s13073-020-00734-5

**Published:** 2020-04-29

**Authors:** Elliott Rees, Michael J. Owen

**Affiliations:** MRC Centre for Neuropsychiatric Genetics and Genomics, Neuroscience and Mental Health Research Institute and Division of Psychological Medicine and Clinical Neuroscience, Cardiff University, Hadyn Ellis Building, Maindy Road, Cardiff, CF24 4HQ UK

**Keywords:** Precision psychiatry, Schizophrenia, Genetics, Genomic risk scores

## Abstract

The primary aim of precision medicine is to tailor healthcare more closely to the needs of individual patients. This requires progress in two areas: the development of more precise treatments and the ability to identify patients or groups of patients in the clinic for whom such treatments are likely to be the most effective. There is widespread optimism that advances in genomics will facilitate both of these endeavors. It can be argued that of all medical specialties psychiatry has most to gain in these respects, given its current reliance on syndromic diagnoses, the minimal foundation of existing mechanistic knowledge, and the substantial heritability of psychiatric phenotypes. Here, we review recent advances in psychiatric genomics and assess the likely impact of these findings on attempts to develop precision psychiatry. Emerging findings indicate a high degree of polygenicity and that genetic risk maps poorly onto the diagnostic categories used in the clinic. The highly polygenic and pleiotropic nature of psychiatric genetics will impact attempts to use genomic data for prediction and risk stratification, and also poses substantial challenges for conventional approaches to gaining biological insights from genetic findings. While there are many challenges to overcome, genomics is building an empirical platform upon which psychiatry can now progress towards better understanding of disease mechanisms, better treatments, and better ways of targeting treatments to the patients most likely to benefit, thus paving the way for precision psychiatry.

## Background

The past decade has seen extensive advances in psychiatric genomics in disorders such as schizophrenia, bipolar disorder (BD), major depressive disorder (MDD), autism spectrum disorder (ASD), attention-deficit hyperactivity disorder (ADHD), intellectual disability (ID), and anorexia nervosa (Table 1). Recent genetic studies of psychiatric disorders have generally adopted one of the following genome-wide approaches: (1) genome-wide association studies (GWAS), which compare the frequencies of single-nucleotide polymorphisms (SNPs) between cases and controls. GWAS do not usually identify causal mutations, but instead identify SNPs in high linkage disequilibrium with the risk variant, thus highlighting genomic regions containing common risk alleles. The aggregated risk from common alleles across the genome is often summarized as per-individual genomic risk scores (GRSs; also known as polygenic risk scores (PRS)). (2) Rare copy number variant (CNV) studies, which identify sub-microscopic deletions and duplications of DNA associated with traits or diseases. (3) Next-generation sequencing (NGS) studies, which also allow discovery of other rare variants such as rare single-nucleotide variants (SNVs) and small insertions/deletions (indels). In psychiatry, most NGS studies to date have focused on sequencing the protein-coding regions of the genome, known as exome sequencing, to identify specific genes or sets of genes enriched for rare variants.

**Table 1:** Core symptoms and common characteristics of psychiatric disorders. Ticks and crosses correspond to characteristics commonly observed or absent in the given diagnosis, respectively

Diagnosis	Symptoms	Common characteristics				
		Psychosis	Depression	Cognitive deficits	Childhood onset	Late adolescence/early adulthood onset
Intellectual disability	Severe deficits in intellectual functioning and adaptive skills, usually established with an IQ score < 70.	✘	✘	✓	✓	✘
Autism spectrum disorder	Developmental disorders with abnormal verbal and nonverbal communication and social interaction, and repetitive behaviors. Symptoms range from moderate to severe.	✘	✘	✓	✓	✘
Attention-deficit hyperactivity disorder	Persistent patterns of inattention and/or hyperactivity, impulsive behavior, which disrupt social, academic or occupational functioning.	✘	✘	✘	✓	✘
Schizophrenia	Positive (hallucinations, delusions), negative (diminished emotional expression), cognitive decline, disorganized thinking.	✓	✘	✓	✘	✓
Bipolar disorder	Episodes of extreme high (mania) and low (depression) moods.	✓	✓	✘	✘	✓
Major depressive disorder	Persistent feelings of sadness and hopelessness. Inability to concentrate and diminished interest in most activities.	✘	✓	✘	✘	✓

The findings that have emerged from these studies have established the genetic architecture of psychiatric disorders as highly polygenic; hundreds to thousands of risk alleles are spread widely across the genome. The population frequencies of these alleles, in part, reflect the degree to which they increase the risk for developing a psychiatric disorder; high-risk variants have lower allele frequencies as they are quickly removed from the population by natural selection [1, 2]. Genetic studies of psychiatric disorders have also shown that pleiotropy (i.e., variants associated with multiple traits) is widespread among risk alleles. However, psychiatric disorders are multifactorial, with environmental exposures also contributing to their development, such as obstetric complications, early-life adversities, migration, and substance abuse [3].

In this review, we will address the question of how recent and future genomic discoveries might be used to help us to better understand disease mechanisms in order to develop new treatments and to target current and future treatments to the patients most likely to benefit, thus paving the way for precision psychiatry. We begin by reviewing recent studies that have enhanced our understanding of the genetic architecture of psychiatric disorders, as well as those that have provided insights into their pathophysiology. We then discuss how the complex genetics of psychiatric disorders have impacted progress in precision psychiatry. Finally, we consider the research agenda for translating recent findings in psychiatric genetics towards the development of better treatments.

## Recent advances in neuropsychiatric genomics

Progress has been greater in some disorders than in others, and we are nowhere near the end of the gene discovery road for any disorder. Nonetheless, enough has been revealed to delineate two features of the genetics of these conditions that will have important implications for translation. First, psychiatric disorders are highly polygenic, meaning that individual risk reflects the combined effects of variation at many different genetic loci. Second, there is extensive pleiotropy, with the effects of risk alleles crossing boundaries between diagnostic categories and between those disorders and behavioral traits in non-clinical populations.

### Polygenicity

**Fig. 1:**
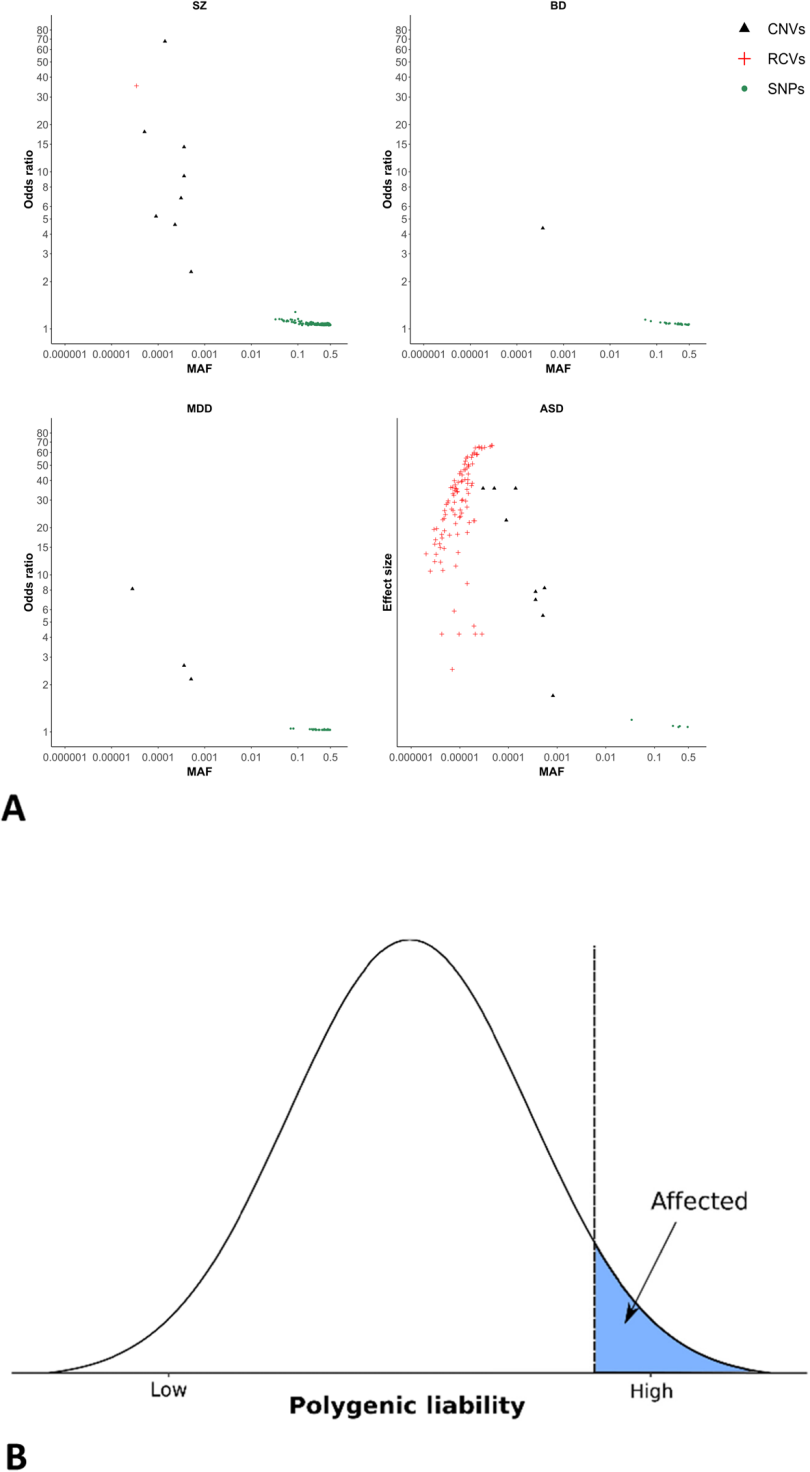
**a** Genetic associations with schizophrenia, bipolar disorder, major depressive disorder, and autism spectrum disorder. Odds ratios (*y*-axis, −log10, transformed to be > 1) and minor allele frequencies (MAF, *x*-axis, −log10, transformed to be ≤ 0.5) for single-nucleotide polymorphisms (SNPs), rare coding variants (RCVs), and rare copy number variants (CNVs), which were derived from the studies outlined in Table 2. We note that for ASD, as odds ratios have not been estimated for all classes of mutation, we have plotted the smoothed relative risk for RCVs (as reported in [14]) and odds ratios for CNVs and SNPs on the same scale, labeled as “Effect sizes,” for illustrative purposes. There is a general trend for a negative correlation between odds ratio and MAF, which reflects the degree to which selection removes risk alleles from the population. **b** Polygenic liability threshold model. For polygenic neuropsychiatric disorders, diverse classes of mutation contribute to liability, with additive models currently providing the best fit to the data [26]

Psychiatric disorders are highly polygenic [Table 2 and Fig. 1a]. Many common alleles of small effect contribute to all the major psychiatric disorders studied to date [7, 10, 13, 16, 27, 28], even developmental disorders (DD) where rare high-penetrance mutations are frequently involved [23, 24]. GWAS have identified hundreds of associations between specific SNPs and psychiatric disorders (Table 2), the majority of which reside in non-coding regions [29, 30]. The greatest progress has been in studies of schizophrenia, where 145 independent loci are implicated in the most recent published GWAS study of 40,675 cases and 64,643 controls [4]. With larger samples, similar progress is likely to be made in other disorders, such as BD and ADHD, given they have similar estimates of SNP-based heritability to schizophrenia (Table 2).

**Table 2:** Summary of genetic findings for neuropsychiatric disorders

**Disorder**	**GWAS**						**Exome sequencing**					**CNVs**				
	**Cases**	**Control**	**GWAS loci**	**Population prevalence (k)**	**Liability-based SNP heritability (SE)**	**Reference**	**Cases**	**Control**	**Trios/quads**	***N*genes**	**Reference**	**Cases**	**Control**	**Trios/quads**	**Loci**	**Reference**
SCZ	40,675	64,643	145	0.007	0.23 (0.0063)	[4]	4264	9343	1077	1	[5]	21,094	20,227		8	[6]
BP	20,352	31,358	17	0.01	0.18 (0.011)	[7]	1201	5233		0	[8]	9129	63,068		1	[9]
ADHD	20,183	35,191	12	0.05	0.22 (0.014)	[10]	4263	5233		0	[8]	896	2455		1	[11]
AN	3495	10,982	1	0.01	0.20 (0.021)	[12]										
ASD	18,381	27,969	5	0.012	0.12 (0.010)	[13]	5556	8809	6430	102	[14]			5574	10	[15]
MDD	135,458	344,901	44	0.15	0.087 (0.004)	[16]						23,979	383,095		3	[17]
OCD	2688	7037	0	0.025	0.28 (0.04)	[18]						1613	1789		1	[19]
TS	4819	9488	0	0.008	0.21 (0.024)	[20]			802	1	[21]	2434	4093		2	[22]
DD	6987	9270	0	0.01	0.077 (0.021)	[23]			7580	94	[24]	29,085	19,584		70	[25]

Common allele loci from genome-wide association studies (GWAS) are reported from the largest SNP-based studies. Individual genes associated with rare coding variants are reported from exome-sequencing studies with > 3000 cases and controls or > 500 proband-parent trios. Loci enriched for rare copy number variants (CNVs) are reported from studies with > 3000 cases and controls. *ADHD* attention-deficit hyperactivity disorder, *AN* anorexia nervosa, *ASD* autism spectrum disorder, *BD* bipolar disorder, *MDD* major depressive disorder, *OCD* obsessive-compulsive disorder, *TS* Tourette syndrome, *SZ* schizophrenia, *DD* developmental disorders, *SE* standard error

Data from genomic microarray and exome sequencing studies have also implicated rare, more highly penetrant mutations [6, 14, 15, 24, 31–34]. The strongest evidence for specific rare variants currently comes from studies of CNVs, where several pathogenic deletions and duplications have been identified [6, 15, 25, 32]. These CNVs are usually large (e.g., disrupting > 500 kb of DNA) and disrupt multiple genes. Only one single-gene CNV, deletions at *NRXN1*, has been robustly associated with schizophrenia [6, 32, 35].

Rare SNVs or indels (hereafter referred to as rare coding variants (RCVs)), and particularly but not exclusively loss-of-function variants in highly conserved genes [36], are enriched in neurodevelopmental disorders (NDDs) such as ID, ASD, ADHD, and schizophrenia. The burden of these mutations is greater in more severe, early-onset disorders such as ID and ASD [37], and there is evidence that stratification exists according to the presence or absence of comorbid ID, with pathogenic CNVs and damaging RCVs being more common in cases with cognitive impairment [38]. To date, RCVs have been associated with 102 and 94 genes in ASD [14] and DD [24], respectively. The majority of these genes are associated with LoF variants, with only four genes (*SLC6A1*, *DEAF1*, *KCNQ3*, and *SCN1A*) preferentially associated with missense variants in ASD [14]. In sequencing studies of schizophrenia, three genes (*SETD1A*, *RBM12*, and *SLC6A1*) have been robustly associated with RCVs [5, 39, 40]; however, the strong excess of LoF variants in constrained genes in schizophrenia suggests that additional single-gene associations will emerge from larger NGS studies.

Finally, there is strong evidence emerging that, in cases carrying rare high-penetrance risk alleles, outcomes are to some extent dependent on the burden of common risk alleles [23, 41–44]. Individuals with schizophrenia that carry pathogenic CNVs still have an elevated burden of common risk alleles compared with controls [42]; the burden of common risk alleles in CNV carrying cases is negatively correlated with the effect size of the CNV (i.e., fewer common risk alleles are found in carriers of higher penetrant CNVs) [44]. Schizophrenia patients carrying de novo LoF variants in evolutionary constrained genes also have a lower burden of common risk alleles when compared with non-carrying cases [39]. These observations support a liability threshold model of schizophrenia [26], where fewer common risk alleles are required to develop schizophrenia in carriers of rare, more highly penetrant mutations (Fig. 1b).

### Pleiotropy

The occurrence of pleiotropy for common risk alleles has recently been evaluated in an analysis of publicly available GWAS results from 558 traits, where over 90% of genome-wide significant loci were shown to be pleiotropic [45]. For psychiatric disorders, there is substantial overlap in rare and common risk alleles in a way that points to extensive biological pleiotropy [46]. Thus, the CNVs that are associated with increased risk of schizophrenia are also often associated with ASD, ID and ADHD [47], and every CNV that confers risk of schizophrenia also does so for ID [48]. Moreover, there is overlap between the genes disrupted by de novo RCVs in schizophrenia, ASD and DD [31, 39, 49]. There is also extensive pleiotropy of common allele effects as evidenced by the substantial genetic correlations observed between pairs of psychiatric phenotypes [46, 50] and with a number of behavioral traits [50]. For example, schizophrenia common alleles are significantly correlated with BD, ADHD, anorexia nervosa, MDD, and OCD [50]. This degree of pleiotropy is in contrast to the situation across neurological disorders, where no significant correlations have yet been found, although genetic correlations have been observed between neurological and psychiatric phenotypes (e.g. migraine is correlated with Tourette syndrome and MDD) [50]. A recent cross-disorder meta-analysis of eight psychiatric disorders (schizophrenia, AN, ADHD, ASD,BD, MDD, obsessive-compulsive disorder and Tourette syndrome) found 109 genome-wide significant pleiotropic loci affecting more than one disorder, of which 23 loci were associated with four or more disorders [51]. These observations almost certainly reflect at least in part the syndromic nature of psychiatric diagnoses [52] and support evidence from a variety of sources that current diagnostic categories are not capturing biologically distinct disease entities [52].

### Biological insights

Studies of rare variants have provided replicated evidence for the involvement of synaptic dysfunction in schizophrenia pathogenesis [31, 33, 53]. For example, the activity-regulated cytoskeleton-associated protein (ARC) and *N*-methyl-*D*-aspartate receptor (NMDAR) postsynaptic protein complexes, which are involved in synaptic plasticity, are disrupted by de novo CNVs in schizophrenia [53]. These synaptic genes have also been associated with schizophrenia in de novo and case-control RCV studies [31, 33, 54]. However, an excess of RCVs in schizophrenia exists more broadly across thousands of neuronally expressed genes, in particular those that are related to the synapse [33]. Common allele studies of schizophrenia have also implicated genes involved in calcium signaling and glutamatergic neurotransmission [29], the latter showing convergence with rare variant studies in supporting a role for synaptic plasticity.

Genes that regulate transcriptional activity have been strongly associated with RCVs in several psychiatric disorders; for example, *CHD8* and *SETD1A*, which are involved in chromatin remodeling, are associated with LoF variants in early-onset NDDs (ASD and DD) and schizophrenia, respectively [5, 14, 24]. Moreover, leukocyte transcriptomic data from children with ASD and controls has shown genes differentially expressed in ASD are directly targeted by transcriptional regulators implicated in ASD (e.g. *CHD8* and *FMR1*), and are indirectly targeted by rare ASD risk alleles that perturb signaling pathways, such as the RAS–ERK, PI3K–AKT and WNT–β-catenin pathways [55]. Transcriptomic data have also highlighted the spatiotemporal expression patterns of ASD risk genes; risk genes involved in regulating gene activity are preferentially expressed in early fetal development, whereas risk genes involved in neuronal communication are most highly expressed in late fetal and perinatal development [14].

The large number of loci, genes and gene sets that continue to emerge from studies of psychiatric disorders makes it challenging to derive specific biological insights from genetic associations. However, single-cell RNA sequencing studies have started to identify cells and tissues mapping to loci implicated from genetic studies. For example, single-nuclei RNA sequencing of cortical tissue from post-mortem brains found genes to be differentially expressed within upper-layer cortical neurons and microglia in ASD [56]. Single-cell RNA sequencing has also been used to map schizophrenia common risk alleles to genes preferentially expressed in pyramidal cells, medium spiny neurons and interneurons [57].

## Implications of genomic findings for progress towards precision psychiatry

**Fig. 2:**
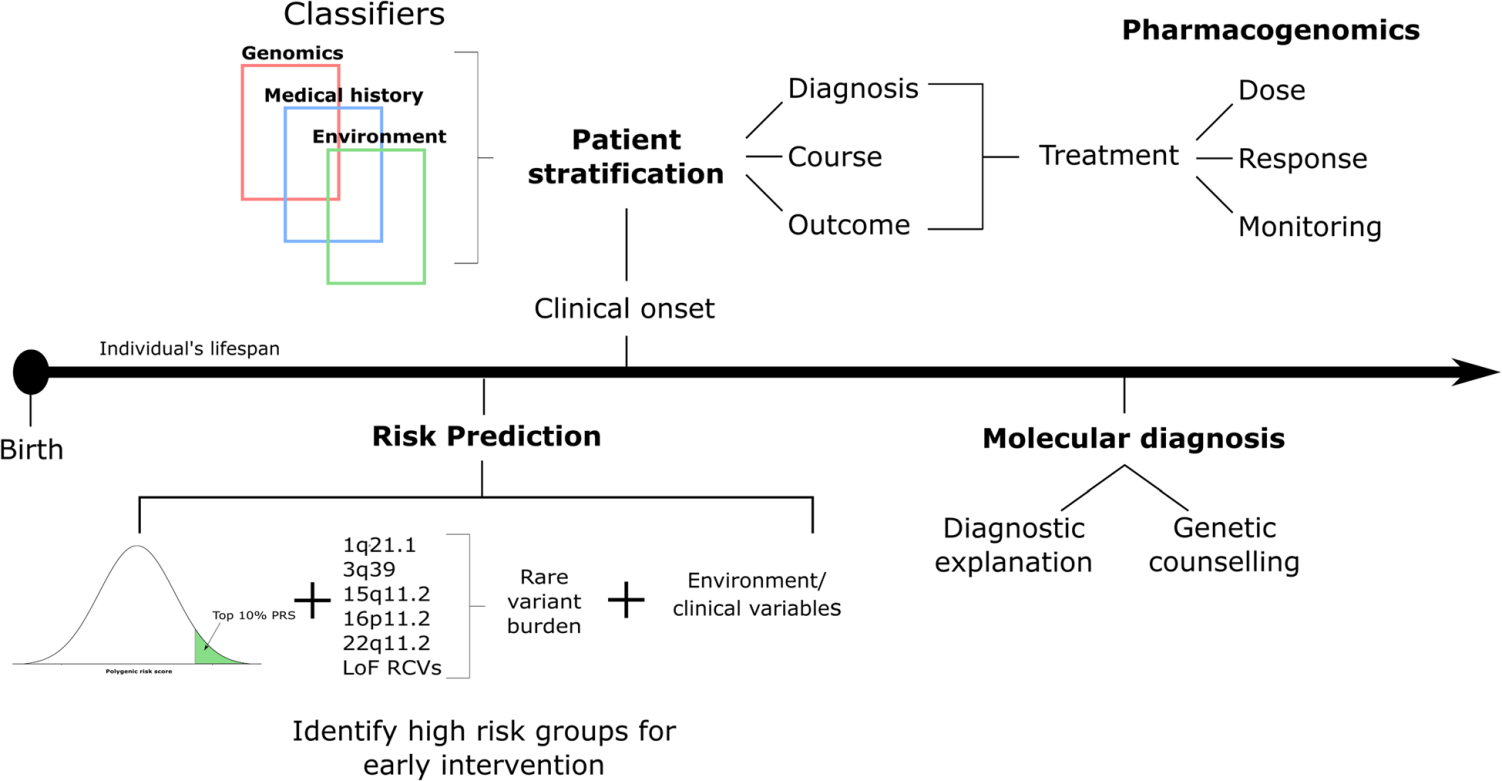
Illustration of what precision psychiatry might look like. With the increasing use of high-throughput genomic technologies in clinical genetics (e.g., the 100 K Genomes Project), it is likely that genomics will eventually have a role in psychiatric healthcare. Quantitative measures of the genetic liability to psychiatric disorders, such as polygenic risk scores, could be combined with additional clinical variables and psycho-social risk factors to help tailor treatment to the individual at several junctures across the lifespan. We briefly highlight here four key areas (risk prediction, patient stratification, pharmacogenomic and molecular diagnostics) where precision psychiatry would directly benefit the management and treatment of patients and provide a general timeline for when they could impact healthcare across an individual’s lifespan (Fig. 2). Risk prediction: Genetic risk scores could help target early intervention strategies towards those at greatest risk for developing a major psychiatric disorder. For example, schizophrenia genomic risk scores could be used to help predict which individuals from phenotypically defined high-risk groups are more likely to develop psychosis. Moreover, individuals who carry a pathogenic copy number variant could receive additional monitoring and/or screening for psychiatric and/or physical comorbidities. Patient stratification: Psychiatric disorders are associated with marked clinical variability in disease course and outcome, both within and across diagnostic categories. Research into biological and environmental exposures associated with this variability will inform stratification of patients into those that could benefit from tailored programs of treatment. Pharmacogenomics: Pharmacogenomic variants are known to influence variation in drug response. Precision psychiatry could therefore impact the way drugs are prescribed, by identifying patients most likely to benefit, predicting the dose required to maximize their therapeutic effects, and identifying patients who require additional monitoring for adverse side effects. Molecular diagnosis: No individual genetic variant is either necessary or sufficient to cause psychiatric disorders; however, the identification of rare, highly penetrant risk mutations, such as 22q11.2 deletions, can help towards providing a diagnostic explanation for the development of a psychiatric disorder. As our knowledge about the penetrance and phenotypic variability associated with rare risk variants improves, their identification among psychiatric patients will inform both genetic counseling and the examination of comorbidities

The primary aim of precision medicine is to tailor healthcare to individual patients (Fig. 2). This will require progress on two broad fronts. First, we need to be able to define patients or, more realistically, groups of patients for whom a particular treatment or other intervention is warranted. Second, we need to develop and test novel treatments and interventions that can be applied with a degree of specificity to individual patients or groups of patients. Indeed, a major justification for the pursuit of psychiatric genomics is that it offers a potentially unbiased route into understanding pathogenesis, and, as a consequence, the promise of new, rationally designed and targeted treatments. The emerging findings from psychiatric genomics have implications for both of these and we will consider them in turn.

### Clinical stratification

The extensive polygenicity and pleiotropy, which seem to be the rule rather than the exception in psychiatric disorders, have important implications for future attempts to use genomic testing in precision psychiatry to define patients or groups of patients for clinical purposes [58, 59]. For example, first, not all individuals with a particular psychiatric disorder will carry the risk allele at any particular locus. Second, unaffected individuals will carry multiple risk loci, with the outcomes associated with any particular risk allele, even high-penetrance alleles, depending on the genetic and environmental context and possibly chance [23, 28]. Third, there will be many different combinations of risk alleles in different cases. Fourth, individuals with different psychiatric outcomes will share a proportion of their underlying genetic risk [48, 50].

Thus, the relationship between psychiatric disorders and the genome is highly complex and far from a simple one to one mapping. This is apparent even when we consider the relatively simple scenario provided by risk CNVs. For example, at least eight distinct CNVs are robustly associated with schizophrenia [6, 32, 48]. However, these display a combination of incomplete penetrance and variable expressivity. For instance all schizophrenia risk CNVs are also associated with ID and many have been associated with a broad range of other psychiatric, cognitive, motor and physical disease outcomes [15, 17, 48, 60–62]. The situation with regard to the large number of alleles of small effect is likely to be even more complex and, at least with the majority of currently used psychiatric and behavioral phenotypes, is unlikely to be significantly simplified by the use of endophenotypes (i.e., measures of pathology that are heritable, associated with the disorder, and observed in seemingly unaffected individuals) as these appear to display similarly complex genetic architectures (see below).

Broadly speaking, there are several headline implications of these complexities for the potential of genomics to inform stratification in precision psychiatry. First, we will have to deal with genomic risk that is spread across many loci in the genome. Second, genomic profiles alone are unlikely to delineate circumscribed categories of patients suitable for specific interventions. Third, genomic profiling in clinical settings is likely to cut across current diagnostic categories, psychiatric sub-specialties and other medical specialties. However, genomics may have the potential to indicate the probability that an individual will fall into a particular clinically relevant group, or for patients carrying rare, high-penetrant mutations (e.g. CNVs), inform screening for additional comorbidities.

### Risk prediction

Common risk alleles are estimated to account for at least a third of the liability to schizophrenia [29], although only 6% of this is explained in the largest GWAS published to date [4]. Although schizophrenia PRS has been robustly associated with increased risk for schizophrenia in real-world clinical settings [63], the predictive ability of schizophrenia PRS alone is at present too low to be informative for an individual. However, these approaches have shown promise in stratifying risk for non-psychiatric traits that are polygenic. For example, in coronary artery disease (CAD), up to 8% of the population have been shown to have CAD GRS that confers a level of risk equivalent to known monogenic rare variants [64]. Risk prediction will also benefit from combining GRS with other risk factors, such as age, environmental exposures and biomarkers; this has been shown in studies of prostate cancer, where prediction models based on prostate-specific antigen alone were significantly improved when combined with GRSs and clinical variables (e.g. age, family history, previous prostate biopsy and prostate exam) [65].

Researchers have begun to evaluate the clinical utility of GRS for predicting diagnostic outcomes and response to treatment in persons meeting research criteria for psychosis high risk and patients with first-episode psychosis. Here, schizophrenia PRS has been shown to be significantly elevated in individuals that later developed schizophrenia compared with those that did not [66, 67], and there is evidence that patients with lower schizophrenia PRS respond more positively to antipsychotic treatment [68]. However, the discriminative accuracy of GRS for predicting outcome and treatment response is at present too low to be clinically useful [66].

### The challenges for mechanistic research

The development of novel, rationally designed treatments will require us to gain new mechanistic insights from genomic and other data. Psychiatric phenotypes are complex and difficult to assess objectively and, as we have seen, most current diagnostic categories are syndromic. The human brain is by several orders of magnitude the most complex organ in the body, and it remains poorly understood and relatively inaccessible to direct analysis. It follows that, given the resulting low base of existing mechanistic understanding coupled with the substantial heritability of psychiatric disorders estimated from genetic epidemiology [69], psychiatry potentially has the most to gain of any branch of medicine from genomic insights into disease biology and the identification of novel drug targets. However, the emerging picture from genomic studies points to a number of key challenges.

First, GWAS do not always directly identify risk variants or risk genes, and the large number of loci implicated and low individual effect sizes do not lend themselves to the canonical functional approaches that are typically applied to Mendelian disorders. There are however grounds for optimism given the increasing availability of sequence-based, functional genomic approaches that integrate genomic data with functional annotations of the genome [70]. These approaches, which allow functional annotation at genomic scale and the potential ability to identify the casual variants underlying SNP associations, are increasingly being applied to psychiatric disorders. This work has been supported by initiatives such as CommonMind and PsychENCODE, which provide transcriptomic and epigenomic data from psychiatric cases and controls for the research community [71–73]. These resources have been used to gain novel mechanistic insights into disease pathogenesis through the development of transcriptome-wide association studies (TWAS), which use methods such as expression quantitative trait loci (eQTL) analysis to refine risk loci to genes, cells and tissues [74]. Recent schizophrenia TWAS have identified correlations between GWAS signals and the expression of 256 genes across 13 different brain regions, with the dorsolateral prefrontal cortex (DLPFC) showing the largest number of significant genes [75]. Another TWAS that focused specifically on genes expressed in the DLPFC found schizophrenia GWAS loci to be associated with the expression of 89 genes, with no significant bias for these genes being preferentially over or under-expressed [76]. Gene-set analyses have also found TWAS associated genes to be enriched among FMRP targets [75], as well as genes related to ‘abnormal central nervous system synaptic transmission’ and ‘antigen processing and presentation of peptide antigen via MHC class I’ [76]. Although TWAS of ASD and BD are currently less powered compared with schizophrenia, recent findings have indicated that TWAS associated genes are shared across these disorders [77].

GWAS and TWAS have also highlighted putative candidates for drug repurposing. For example, schizophrenia GWAS studies have implicated genes that are targets of compounds with approved indications, such as voltage-gated calcium channels (e.g. CACNA1C, CACNB2 and CACNA1L); pharmaceutical compounds that are antagonists or activators for these calcium channels have been approved for the treatment hypertension, arrhythmia or epilepsy [78]. Comparisons between drug induced and disease-associated gene expression profiles have also identified correlations between known psychiatric indications. For example, antipsychotic induced expression profiles are correlated with GWAS imputed gene expression profiles for schizophrenia and bipolar disorder [79]. Correlations between drug induced and GWAS imputed gene expression profiles could therefore be used to prioritize candidates for novel drug repositioning.

However, challenges remain in psychiatry due to the complexity of the brain compared to other organs and the possibility that pathogenic mechanisms might operate over long developmental periods.

Second, recent genomic findings have implications for human mechanistic studies that attempt to link risk factors and clinical phenotypes via underlying pathophysiology and mechanisms using so-called endophenotypes [80], such as measures of cognition, brain structure, electrophysiology and biochemistry. The assumption that endophenotypes are in general likely to be less complex genetically than clinical disorders has been questioned [81]. More importantly, the possibility of pleiotropy implies the need for caution before concluding that a particular endophenotype mediates the effect of risk on clinical phenotypes [46, 82]. For example, there is strong evidence that the burden of RCVs in schizophrenia is greatest in patients with comorbid ID, but RCVs are still enriched in schizophrenia patients without ID [38]. Therefore, the cognitive impairments associated with rare variants that confer risk to psychiatric disorders do not completely mediate the risk of psychopathology [46]. The study of endophenotypes does however offer a promising means by which genetic risk can be linked to disturbances of brain function [83], and may help to close the gap between genes and behavior assuming that the results are interpreted with sufficient caution.

Third, the degree to which a very large number of common variants with low effect sizes confer risk to psychiatric disorders poses challenges for using genomic discoveries to develop animal and cellular models with high construct validity (that is, that can adequately model a psychiatric disorder). The function of individual genes implicated by GWAS can be explored in animal models, but it is hard to see how large numbers of human risk alleles can be modeled with current or emerging technologies, even supposing that a sufficient number of causal variants can be identified. It is possible to model rare variants associated with higher risk including CNVs and RCVs in rodents [84], thus allowing behavioral and network studies as well as molecular analyses, and the potential of such approaches has been enhanced by the development of genome engineering approaches such as CRISPR-based technologies [85]. But even here, the degree of variable expressivity and pleiotropy seen in human carriers are grounds for caution in drawing causal inferences between endophenotypes and behavior, as is the case in human studies (see above) [46], and for many psychiatric disorders the number of individual genes robustly associated with moderate-to-high-penetrance RCVs or CNV loci has not to date been great (Table 2).

Many of the same considerations apply to cellular models, but here there is potential to model human genetic complexity directly in induced pluripotent stem cells (iPSCs). These can be derived from patients [86] and also target individuals carrying high-penetrance mutations [87] or on the basis of genomic risk scores or a combination of the two. Notwithstanding the increasing interest in brain organoids [88], these approaches are currently limited in complexity and to early stages of development (see below).

## The research agenda for translating insights from genetics to precision psychiatry

Having summarized the advances in psychiatric genomics and the broad challenges facing translation of these findings, we now consider the priorities for research aiming to build on these advances to obtain better treatments and to target these effectively. Though we consider them separately, we note that there will need to be much cross-talk between the different areas of research given the limitations associated with each.

### Risk prediction

Various methods have been developed to quantify risk, heritability and genetic correlation from common alleles [58]. The power of these approaches is increasing as data from larger GWAS become available, though in the case of psychiatric disorders, these methods remain underpowered to be used in population screening. The predictive utility of such testing will depend on the risk profile in the group to which the testing is being targeted, as well as the availability of effective preventative measures that can be targeted to the high-risk groups. This will require further research, and one area in psychiatry that is sure to receive attention is the ability of GRS to inform management in first-episode psychosis, given that the outcomes of patients are highly variable following treatment. Another important current limitation of the clinical use of GRS is the lack of ancestral diversity in GWAS studies, the vast majority of which have been carried out in individuals with European ancestry [89]. Significantly less heritable variation is explained when GRSs are applied trans-ethnically [89] and it will require well-powered GWASs to be undertaken in many diverse populations to avoid further exacerbation of current global health disparities [89].

Heritability estimates for psychiatric disorders from pedigrees are substantially larger than SNP-based heritability; for example, schizophrenia pedigree and SNP-based heritability estimates are ~ 80% [90] and 23% [4], respectively. This difference in heritability is, in part, due to rare variants that are not tagged on SNP arrays. Indeed, the importance of accounting for the full frequency distribution of alleles contributing to a polygenic trait has recently been exemplified in a study of height and BMI, which used whole genome sequencing (WGS) to show that heritability estimates from molecular genetic data aligned with those from family and twin studies when both rare and common alleles were considered [91].

A number of rare variants have been identified that confer risk to psychiatric disorders including both CNVs and RCVs (Table 2 and Fig. 1a), and many more are likely to be discovered in future large-scale sequencing studies. As the number of rare variants associated with psychiatric disorders increases, the use of WGS has great potential to improve risk prediction/stratification accuracy, as the upper limit of prediction is the heritability; however, increasing the number of alleles used for out-of-sample prediction can reduce the expected proportion of variance explained, and therefore, massive samples are needed for WGS to enhance prediction accuracy [92].

Identification of CNVs through chromosomal microarrays (CMA) is increasingly becoming part of routine clinical testing for ID, DD, and ASD [93] and is currently considered the first-tier test for undiagnosed NDDs. However, there is a strong case for exome sequencing to replace CMA as a first-tier test, given evidence that it provides a higher diagnostic yield for NDDs (36%, compared with 15–20% from CMA) [94]. With the continued reduction in the cost of sequencing, it also seems highly likely that whole genome sequencing will increasingly be applied in these conditions to detect pathogenic RCVs. There have also been calls for extending CMA testing to schizophrenia [95]. Currently, such tests may offer patients and families a degree of diagnostic explanation and also point to the need for genetic counseling as well as indicating increased risk for associated physical comorbidities. However, these tests do not currently offer much in the way of predictive information about psychiatric or behavioral outcomes or information of direct therapeutic relevance. It will be challenging to acquire informative data in these areas given the high degree of pleiotropy and the rarity of many of the pathogenic mutations, and this will require coordinated data sharing across many centers along the lines of the DECIPHER database for DD in the UK [96].

### Identifying novel strata for precision medicine

The aim of precision medicine is to tailor healthcare, including prevention and decisions on treatment and care pathways, more effectively to the needs of individual patients. For this to succeed in psychiatry, it is clear that we need new ways of characterizing patients or groups of patients according to attributes that will allow healthcare to be targeted more effectively. There is now a pressing need to understand how and to what extent genomic variation underlies the variability in symptoms, treatment response, course, and outcome seen in psychiatric disorders, and to determine whether genomic data can contribute to the development of clinically useful strata. For the reasons of polygenicity and pleiotropy outlined above, it seems unlikely that genomics alone alongside current diagnostic categories will satisfy this criterion, though this is a matter for empirical enquiry. Rather, it seems likely that the potential of genomic profiling in precision psychiatry to provide clinically actionable information such as the likelihood of response to a particular drug or other intervention will be its use in concert with other clinical features such as specific symptom profiles, developmental profiles, cognitive, imaging, and other biomarkers.

This is a crucial area if we are to translate genomic discovery into patient benefit, but its potential will only be realized if we develop sufficiently large-scale datasets in which genomic data can be analyzed together with informative phenotypic data. Many of the samples used for psychiatric GWAS have not been phenotyped at depth, and when they have, divergent methods have been employed across sites. There is a need for a major effort here to collate and collect large richly phenotyped samples, to coordinate phenotyping across sites and make the data available to other researchers. Current and emerging population studies and cohorts can play a role in this but typically mental health data are scanty in such samples and for various reasons severe psychiatric disorders such as schizophrenia and childhood NDDs tend to be underrepresented in such studies. There is also huge potential to leverage advances in “Big Data” approaches including access to electronic health records and digital phenotyping [97].

A major challenge facing precision psychiatry is the huge variability in disease course and outcome seen within our currently imprecise diagnostic categories. It is unclear to what extent this reflects diagnostic heterogeneity, pharmacogenetic factors, environmental exposures, or a host of potential factors influencing individual differences impacting behavior. What is clear is that if we are to develop effective precision psychiatry, we need to identify the factors that predict disease course and outcome and this will require suitably powered datasets that capture clinical data over extended time periods. It is hard to see how this can be achieved without access to routinely collected clinical data.

Access to such data is important for a second reason. The great majority of genomic studies aim to identify genetic variants associated with the occurrence of disease. Such variants may well have utility in informing disease prevention, but it may be the case that genes involved in disease progression might be of greater relevance to the development of therapeutic strategies [98].

There is thus a need for more genomic studies of disease course and outcome (Fig. 2). These have been scarce due in part to difficulties in defining progression variables, especially in psychiatry, and there are potential biases that need to be taken into account [98]. However, through ongoing efforts to link large genetic datasets to electronic health records, thus providing longitudinal phenotypic data, we expect to see progress in this area over the coming years.

Another area in which we can expect progress in the application of genomics to precision psychiatry is in pharmacogenomics aiming to identify patient groups that are more or less likely to respond to particular drugs or to develop important adverse effects (Fig. 2). To date, most interest has been focused upon pharmacogenetic evidence implicating the genes encoding known drug metabolizing enzymes, and the HLA system in determining risk to certain adverse effects [99] and, for example, promising findings have recently emerged in relation to risperidone dosage and drug metabolism [100, 101]. However, genomics offers the prospect of undertaking wider, unbiased, searches. Here, the pressing need is for sufficiently powered samples containing reliable and valid data on drug response and adverse effects. There are however promising signs of progress. For example, there is emerging evidence that genetic architecture of susceptibility to schizophrenia may be distinct from that of treatment outcomes [102], and progress is being made in identifying common genetic variants that have large effects on the metabolism of clozapine and its metabolites, opening the way for clinical studies assessing the use of pharmacogenomics in the clinical management of patients with treatment-resistant schizophrenia [101]. In addition, a recent genomic study has indicated that the high rates of neutropenia seen in African ancestry individuals taking clozapine is due to the high frequency of the Duffy-null genotype (Duffy is a red blood cell glycoprotein) in these individuals rather than to the toxic effects of clozapine, which require discontinuation of treatment [103]. The neutropenia associated with the Duffy-null genotype is benign, and these findings suggest that simple genotyping at the locus may allow a rapid diagnosis of “benign ethnic neutropenia” and ensure that many individuals of African ancestry who require clozapine treatment are able to continue treatment despite the presence of neutropenia.

Advances in genomic medicine, in particular for disorders where clinically actionable gene-drug associations exist, have prompted the development of online public databases that summarize published pharmacogenomic findings. For example, PharmGKB [104] provides a manually curated knowledgebase for the research community about genetic variants that influence differences in drug efficacy, dose, and toxicity/adverse drug reactions. The disorders and phenotypes in PharmGKB are comprehensive, and over 150 variant-drug associations are available for schizophrenia; however, many of these associations are derived from small and/or candidate gene studies and thus would not meet accepted standards for robust evidence. An advantage of PharmGKB is that for each drug-variant association, a level of evidence score is provided based on statistical evidence and reproducibility, although these “evidence levels” may not always align with those of the genomics research community. The PharmGKB interface allows for users to filter drug-variant associations for important evidence criteria, such as sample size and *p* values, as well as providing links to primary publications, thus allowing users to make their own assessment of the evidence for a drug-variant association. For example, the largest drug-variant study for schizophrenia in PharmGKB (*N* samples = 8133) reports a common allele on chromosome 12 (rs149104283) associated with clozapine-induced neutropenia (odds ratio (OR) = 4.32, *P* = 1.79 × 10^− 8^) [105]. In summary, databases such as PharmGKB provide a useful resource for the research community, but the associations reported should be treated with caution and users should conduct their own assessment of the level of evidence presented for drug-variant associations.

### Population studies

One of the corollaries of polygenicity is that genetic risk is spread across the population. This provides an important opportunity to undertake research into the impact of genetic risk in cohorts and epidemiological samples taken from the general population. To the extent that this may involve individuals without the disease in question, this mitigates the risk of reverse causation and drug effects, though other sources of confounding may not be excluded [106]. Samples such as the UK Biobank, the ALSPAC birth cohort, the ABCD study, and those available to the Enigma neuroimaging consortium are currently being explored in relation to genetic risk for psychiatric disorders [17, 107–111]. These studies can help identify endophenotypic markers of risk such as brain structure [112, 113] and cognitive impairment [62, 114] and developmental antecedents [115]. Population studies of this sort, especially when combined with the functional genomic approaches described below, offer windows into understanding disease risk mechanisms and how they manifest over the lifespan. Such insights when applied to clinical populations are likely to also impact on stratification.

### Understanding disease mechanisms

As we have seen, the complex genetic architecture of psychiatric disorders poses many challenges for attempts to translate genomics into mechanistic insights. Many of these challenges and potential solutions have recently been reviewed in detail by others [71, 116] and readers are referred to these articles for detailed treatments of the issues, and the methods and resources available.

Broadly speaking, we can define the priority areas of research as follows. First, since most common genetic variation contributing to psychiatric disorders lies outside protein-coding regions, a major effort will be required to understand which genes are impacted by risk variants and the direction of effect. This will require the functional annotation of regulatory regions at a genome-wide scale. Moreover, the size of sequencing studies will increase significantly over the coming years, which will provide powerful approaches for identifying specific target genes associated with rare coding variants. Second, genes do not operate in isolation but rather as parts of networks or pathways, and understanding how polygenic inheritance impacts on such networks and identifying whether risk genes converge on sets of common networks is another major effort that is underway. Third, there is a need to drill down into the question of whether genetic risk operates in specific neurons or groups of neurons and to address the issue of whether risk impacts on the brain at particular points in development. Work across these areas is intensive and is developing rapidly [71, 116], and despite the complexity, there have been some promising findings indicating convergence onto genes implicated in neuronal communication, especially synaptic genes, and regulation of gene expression, including transcription factors and chromatin modification gene sets [5, 14, 31, 53, 117]. Finally, while we can expect many findings to be published addressing these issues in the next 5 years, it is hard to avoid the conclusion that their applicability to precision psychiatry will be limited if they are exclusively anchored to existing syndromic diagnoses. Ultimately, if we are to understand how putative disease mechanisms impact on outcome or response to treatments, we will need to take a more diagnostically agnostic approach and begin to relate such mechanisms to sufficiently fine-grained phenotypic data in most of the large datasets subjected to genomic analysis.

### Model systems

The complex and pleiotropic genetic architecture of psychiatric disorders pose great challenges for work in model systems as we have seen. There is scope to continue developing animal models based upon high-penetrance RCVs or CNVs. However, given the polygenic basis of psychiatric disorders, it seems increasingly unlikely that simple pathways from gene to biology to behavior will be found. It may be possible to use interventional methods such as optogenetics [118] to establish causative pathways between genes and behavior. There will also be a need to model developmental effects and the variability of phenotypic outcomes (genetic background, environmental exposures). There are also well-articulated concerns about the face validity of animal models, particularly of behavior, in psychiatric research [119].

Given advances in stem cell technology it seems likely that there will be a growth of studies using cellular models, which can accommodate genetic complexity by taking cells from patients or high-risk individuals and be subject to experimental manipulation and drug testing. iPSC models have been applied to a range of psychiatric disorders including schizophrenia, bipolar disorder, and Rett syndrome [120]. However, there are intrinsic caveats associated with these approaches that include artifactual heterogeneity, due to factors such as somatic mosaicism in donor cells and de novo mutations occurring during cell reprogramming, loss of epigenetic modifications, and the developmental immaturity of iPSC-derived neurons [121]. Other issues that will need to be addressed are the question of which cell types should be studied in modeling particular disorders and this may be informed by functional genomic studies linking human genomic data with single-cell RNA sequencing datasets [14, 57].

There is also burgeoning interest in the potential of brain organoids to provide better models of brain development and brain diseases than 2D iPSC models [88]. This is a rapidly developing field that is still in the early phases of development and consequently a number of challenges remain to be addressed [88]. The limitations intrinsic to simpler iPSC models still apply but organoids potentially provide an approach whereby the effects of genetic risk on complex cellular interactions can be modeled in human tissue. Since organoids mature slowly, these approaches may be best applied to modeling disease phenotypes that manifest at early stages of development and encouraging findings have been reported in severe neurodevelopmental disorders such as microcephaly, lissencephaly, and Timothy syndrome [88]. Whether they will be informative for disorders which manifest later in life remains to be seen, though encouraging findings have been reported in Alzheimer disease [122].

## Conclusions

The many genomic advances that have been made over the past 20 years have taken us from a state of almost total ignorance as to the biological underpinnings of psychiatric disorders to an appreciation of the underlying complexity, the identification of a large number of risk factors and loci, and the realization that genetic risk maps poorly onto the diagnostic categories that we use in the clinic. In spite of this success and the multitude of new findings, there is more genetics research needed. Much of the heritability of psychiatric disorders remains unexplained by common SNPs and rare mutations, and there is emerging evidence implicating rare missense and rare non-coding variants that will require whole genome sequencing in large samples [14, 39, 91, 123]. The power and utility of genomic approaches to enable risk prediction and stratification will increase as genomic data accumulate, and further benefit will accrue from efforts to integrate genomic data with more fine-grained and longitudinal phenotypic data. There is potential for current genomic findings to have an immediate impact on psychiatry; for example, testing rare pathogenic CNVs in patients would inform screening for comorbidities, and genotyping the Duffy-null allele in patients of African ancestry would inform clozapine treatment. The highly polygenic nature of psychiatric disorders poses challenges for conventional approaches in moving from genes to biology. The hope is that combining genetics, functional genomics and single-cell RNA sequencing will identify convergence onto specific systems, cell types, circuits, developmental stages, etc. There is much to do here using in vivo, in vitro, and increasingly, in silico approaches. As large-scale omics studies continue to elucidate the molecular basis of psychiatric disorders, it is likely that integrating different types of omics data produced from the same samples will enhance approaches to define biologically meaningful subtypes of patients. Identifying patient subtypes that differ with regard to disease progression, clinical outcomes, and treatment response has great potential to enable targeted treatment and improve clinical trials, as has been clearly demonstrated in the field of oncology [124, 125].

While there are certainly challenges to overcome, genomics has built an empirical platform upon which psychiatry can now progress towards better understanding of disease mechanisms, better treatments, and to better ways of targeting treatments to the patients most likely to benefit, thus paving the way for precision psychiatry.

## Data Availability

Not applicable.

## Abbreviations

BD: Bipolar disorder
ASD: Autism spectrum disorder
ADHD: Attention-deficit hyperactivity disorder
ID: Intellectual disability
DD: Developmental disorders
AN: Anorexia nervosa
MDD: Major depressive disorder
OCD: Obsessive-compulsive disorder
TS: Tourette syndrome
SZ: Schizophrenia
RCVs: Rare coding variants
CNVs: Copy number variants
NDDs: Neurodevelopmental disorders
GRS: Genomic risk scores
CMA: Chromosomal microarrays
PRS: Polygenic risk score
CAD: Coronary artery disease
NGS: Next-generation sequencing
eQTL: Expression quantitative trait loci
WGS: Whole-genome sequencing
